# Proteomic analysis of retinal pigment epithelium cells after exposure to UVA radiation

**DOI:** 10.1186/s12886-019-1151-9

**Published:** 2019-08-02

**Authors:** Jiunn-Liang Chen, Chun-Tzu Hung, Joseph Jordan Keller, Hsien-Chung Lin, Yu-Jen Wu

**Affiliations:** 1 Department of Ophthalmology, Kaohsiung Veterans General Hospital, Kaohsiung, Taiwan; 2 School of Medicine, National Yang-Ming University, Taipei, Taiwan; 3 Department of Optometry, Shu-Zen Junior College of Medicine and Management, Kaohsiung, Taiwan; 4 Department of Ophthalmology, Yuan’s General Hospital, Kaohsiung, Taiwan; 5 College of Medicine, The Ohio State University, Columbus, OH USA; 6 School of Public Health, College of Public Health, Taipei Medical University, Taipei, Taiwan; 7 International Master’s Program, College of Health Technology, National Taipei University of Nursing and Health Sciences, Taipei, Taiwan; 8 Department of Ophthalmology, Kaohsiung Medical University Hospital, Kaohsiung, Taiwan; 9 Department of Beauty Science, Meiho University, Pingtung, Taiwan

**Keywords:** Proteomic, UVA, Retinal pigment epithelium cells, Apoptosis, Mitochondrial dysfunction

## Abstract

**Background:**

Age-related macular degeneration (AMD) is the primary cause of blindness and severe vision loss in developed countries and is responsible for 8.7% of blindness globally. Ultraviolet radiation can induce DNA breakdown, produce reactive oxygen species, and has been implicated as a risk factor for AMD. This study investigated the effects of UVA radiation on Human retinal pigment epithelial cell (ARPE-19) growth and protein expression.

**Methods:**

ARPE-19 cells were irradiated with a UVA lamp at different doses (5, 10, 20, 30 and 40 J/cm^2^) from 10 cm. Cell viability was determined by MTT assay. Visual inspection was first achieved with inverted light microscopy and then the DeadEnd™ Fluorometric TUNEL System was used to observe nuclear DNA fragmentation. Flow cytometry based-Annexin V-FITC/PI double-staining was used to further quantify cellular viability. Mitochondrial membrane potential was assessed with JC-1 staining. 2D electrophoresis maps of exposed cells were compared to nonexposed cells and gel images analyzed with PDQuest 2-D Analysis Software. Spots with greater than a 1.5-fold difference were selected for LC-MS/MS analysis and some confirmed by western blot. We further investigated whether caspase activation, apoptotic-related mitochondrial proteins, and regulators of ER stress sensors were involved in UVA-induced apoptosis.

**Results:**

We detected 29 differentially expressed proteins (9 up-regulated and 20 down-regulated) in the exposed cells. Some of these proteins such as CALR, GRP78, NPM, Hsp27, PDI, ATP synthase subunit alpha, PRDX1, and GAPDH are associated with anti-proliferation, induction of apoptosis, and oxidative-stress protection. We also detected altered protein expression levels among caspases (caspase 3 and 9) and in the mitochondrial (cytosolic cytochrome *C*, AIF, Mcl-1, Bcl-2, Bcl-xl, Bax, Bad, and p-Bad) and ER stress-related (p-PERK, p-eIF2α, ATF4 and CHOP) apoptotic pathways.

**Conclusions:**

UVA irradiation suppressed the proliferation of ARPE-19 cells in a dose-dependent manner, caused quantitative loses in transmembrane potential (ΔΨm), and induced both early and late apoptosis.

## Background

Age-related macular degeneration (AMD) is the primary cause of blindness and severe vision loss in developed countries and is responsible for 8.7% of blindness globally [1]. While the clinical progression of AMD is not well understood, the degeneration of retinal pigment epithelial (RPE) cells is suspected to be involved in the pathophysiology [2–5]. The exact cause of RPE cell degeneration is not clear. Numerous reports suggest that oxidative stress plays an important role in RPE apoptosis and the development of AMD [6–11], with current therapeutic strategies under investigation employing agents that reduce oxidative damage [12, 13]. Pathogenic mechanisms have been proposed to explain the complex etiology of AMD based on animal studies and cultured RPE cells, however, the molecular pathology is not yet entirely clear [14, 15].

Ultraviolet (UV) radiation is part of the electromagnetic spectrum. Only ultraviolet B (UVB; 280–320 nm) and ultraviolet A (UVA; 320–400 nm) reach the terrestrial surface [16]. Although the cornea, lens, and the vitreous body filter out most of the UV radiation below 400 nm, a portion of the UVA band (315–400 nm) penetrates the retina [17]. Ozone layer depletion, increased outdoor activity, and longer average life spans have contributed to the increased cumulative lifetime exposure of the retina to UV radiation [18]. UV irradiation can cause the production of reactive oxygen species (ROS), cellular changes, DNA damage, and apoptosis in RPE cells [19–24], and has been implicated as a risk factor for AMD. However, the results of studies investigating the association between UV exposure and AMD are inconsistent [19, 25–28].

In this study, comparative proteomic analysis was employed to identify the differential protein expression between ARPE-19 cells exposed to UVA radiation and unexposed control cells by comparing their 2D electrophoresis maps. These results will help clarify that UVA radiation induces damage to RPE cells at the molecular level, thus better elucidating the relationship between UVA radiation and AMD. This knowledge may benefit the development of preventive methods for UVA-induced retinal damage.

## Methods

### Reagents

Cell Extraction Buffer was produced by BioSource International (Camarillo, CA, USA). Dulbecco’s modified Eagle’s medium–F12 (DMEM-F12), fetal bovine serum (FBS) phosphate-buffered saline buffer (PBS), and trypsin-EDTA were manufactured by Biowest (Nuaillé, France). The IPG buffer, protein assay kit 2-D Quant Kit, and IEF strips utilized in this study were produced by GE Healthcare (Buckinghamshire, UK). We used PVDF (polyvinylidenedifluoride) membranes that are commercially available from Millipore (Bellerica, MA, USA). Sigma (St Louis, MO, USA) manufactured the protease inhibitor cocktail, dimethyl-sulfoxide (DMSO), 3-(4,5-Dimethylthiazol-2-yl)-2,5-diphenyltetrazolium bromide (MTT) and goat anti-rabbit or goat anti-mouse horseradish peroxidase conjugated IgG that were used in this study. Biotium (Hayward, CA, USA) and Pharmingen (San Diego, CA, USA) produced our cationic dye JC-1 (5,5,6,6-tetrachloro-1,1,3,3-tetraethylbenzimidazolcarbocyanine iodide) fluorescent kit and the Annexin V-FITC/PI Apoptosis Detection Kit, respectively. Promega (Madison, WI, USA) manufactured the 4′-6-diamidino-2-phenylindole (DAPI) and DeadEnd™ Fluorometric TUNEL fluorescent kits we used, and Pierce Biotechnology (Rockford, IL, USA) produced our ECL Western Blotting Reagent.

### Cell culture

ARPE-19 (ATCC® CRL-2302™), a human retinal pigment epithelial cell line, was grown in DMEM-F12 medium supplemented with 10% (v/v) fetal bovine serum, streptomycin (100 μg/mL), and penicillin (100 units/mL) in a humidified atmosphere (5% CO_2_ at 37 °C). We cultured ARPE-19 cells in 10 cm dishes. All the experiments were conducted in triplicate and then repeated three times to ensure reproducibility.

### Determination of cellular viability following UVA irradiation

A UVA lamp (312 nm, Spectroline Model LF-106 M, EEC) was used as the UVA source. We first investigated the lethal UVA dose for the cells. A total of 1 × 10^5^ cells were plated in 24-well plates and grown to 70% confluency. After removing the medium, the cells were rinsed twice with phosphate-buffered saline (PBS). The monolayer was then covered with 1 mL PBS and the cells irradiated with a UVA lamp at several doses (5, 10, 20, 30 and 40 J/cm^2^) from a distance of 10 cm. The original medium was replaced, and cellular viability was determined by MTT assay. Following a 24 h incubation, MTT solutions were added and the cells were incubated for an additional 3 h at 37 °C before removing the supernatant and adding 100 μL of DMSO. The absorbance was read at 560 nm with a microplate reader. A mock-irradiated control was also prepared using the same procedure without UVA irradiation. The morphological changes of the cells exposed to UVA radiation were observed with inverted light microscopy. To detect the cytotoxic effect of UVA irradiation on the induction of apoptosis, ARPE-19 cells were then exposed to 5 J/cm^2^ and 10 J/cm^2^ UVA radiation, incubated for 6 h, and stained with Annexin V-FITC/PI for flow cytometry analysis. Only the 5 and 10 J/cm^2^ UVA irradiation dosages were used for the TUNEL/DAPI assay because the of 20, 30 and 40 J/cm^2^ UVA irradiation exposures resulted in severe cellular cytotoxicity and death.

### 4′,6-diamidino-2-phenyl iodide (DAPI) and terminal deoxynucleotidyl transferase dUTP nick end labeling (TUNEL) stain

The staining procedure for DAPI/TUNEL has been described previously [29]. ARPE-19 cells (1 × 10^6^ cells/well) were seeded in 12-well plates, irradiated with a UVA lamp at different doses (0, 5, and 10 J/cm^2^), and incubated for 24 h. We used DMSO as a control. The cells were all fixed with a 4% paraformaldehyde-PBS solution for 15 min and stained with DAPI prior to detecting nuclear DNA fragmentation with the DeadEnd™ Fluorometric TUNEL System (Promega, USA). Fluorescent microscopy was utilized to photograph the cells (Olympus IX71 CTS, Chinetek Scientific, Hong Kong, China).

### Mitochondrial membrane potential (ΔΨm) assay using fluorescence microscopy

Mitochondrial membrane potential was assessed with 5, 5′, 6, 6′-tetrachloro-1, 1′, 3, 3’tetraethylbenzimidazolylcarbocyanine iodide (JC-1) according to the manufacturer’s protocol (Biotium, Hayward, CA, USA). ARPE-19 cells (1 × 10^5^ cells/well) cultured in 12-well plates were exposed to UVA irradiation at different doses (control, 5, and 10 J/cm^2^) and grown in a 37 °C incubator for 24 h. Following incubation, we harvested the cells and washed them with PBS before adding 70 μL of JC-1 staining solution and incubating them at 37 °C for 30 min in the dark [30]. The cells were directly observed with a fluorescent microscope (Olympus IX71 CTS, Chinetek Scientific, Hong Kong, China) after washing with PBS to remove unbound JC-1 dye.

### 2-DE analysis

The standard procedure for 2-DE was described in our previous study [31]. Three hundred micrograms of total protein was dissolved in rehydration buffer (8 M urea, 2% CHAPS, 40 mM DTT, 0.5% IPG buffer pH 3–10, and bromophenol blue) and loaded onto an IPG strip in a strip holder. We performed the first dimension electrophoresis at 20 °C on the IPGphor III (GE Healthcare). We rehydrated every 11-cm IPG strip (pI 3–10, Immobiline DryStrip) at 50 V for 12 h, before focusing as follows: 200 V (2 h), 500 V (2 h), 1000 V (2 h), 4000 V (2 h), 8000 V (4 h), until the total Vh reached 43,400 Vh. We then placed the equilibrated strip on a 12.5% SDS-PAGE gel for the second dimension electrophoresis. Visualization was achieved with CBR staining and images were analyzed with PDQuest 2-D Analysis Software (Bio-Rad, USA) for quantification. Spots with more than 1.5-fold difference were selected for further LC-MS/MS analysis.

### Protein digestion and protein identification by LC-MS/MS

The standard procedure for protein identification by LC-MS/MS was described in our previous study [32]. Target protein spots were excised from the gel and allowed to undergo reduction by adding 100 μL of 50 mM DTT/25 mM ammonium bicarbonate (pH 8.5) at 37 °C for 1 h. A further alkylating reaction was then performed through the addition of 100 μL of 100 mM iodoacetamide/25 mM ammonium bicarbonate (pH 8.5) and reacted for 30 min in the dark at room temperature. We added 10 μL of trypsin solution (10 ng/mL in 25 mM ammonium bicarbonate) to the gel, digested the protein for 24 h at 37 °C, and concentrated digestion peptide solution prior to LC-MS/MS analysis. We used an AB SCIEX QTRAP® 5500Q mass spectrometer (Applied Biosystems, CA, USA). The MS scan range was m/z 100 to 1000. Analyst 1.5.1 was used to process the raw data into a WIFF text file.

### Western blot analysis

We purchased antibodies against Heat shock protein 27 (Hsp27), nucleophosmin (NPM), ATP synthase, and GAPDH from Epitomics (Burlingame, CA, USA) and antibodies against glucose-related protein 78 (GRP78), calreticulin (CALR), protein disulfide isomerase (PDI), cleaved-ATF6, ATF4, and PRDX1 antibodies were purchased from ProteinTech Group (Chicago, IL, USA). Antibodies against Bcl-xl, Mcl-1, Bcl-2, Bad, p-Bad, AIF, cytochrome *C*, PERK, p-PERK, eIF2α, p-eIF2α, CHOP, caspase-3, cleaved caspase-3, caspase-9, and cleaved caspase-9 were obtained from Cell Signaling Technology (Danvers, MA, USA). The protein collection and separation are explained elsewhere [33]. The primary antibody with appropriate dilution was incubated with the PVDF membrane at 4 °C for 2 h. The membrane was washed three times with PBST (10 mM NaH_2_PO_4_, 130 mM NaCl, 0.05% Tween 20), and then incubated with the second antibody (goat anti-rabbit or goat anti-mouse IgG and horseradish peroxidase conjugate, 1:5000 in blocking solution) at 4 °C for 2 h. The membrane was then washed three times with PBST. The western blot results were visualized through chemiluminescence by adding ECL Western Blotting Reagents (Pierce).

### Statistical analysis

The results of the MTT assay were analyzed with Student’s t-test (Sigma-Stat 2.0, San Rafael, CA, USA). Results with *p* < 0.05 were considered statistically significant.

## Results

### Cell viability after UVA irradiation

**Fig. 1:**
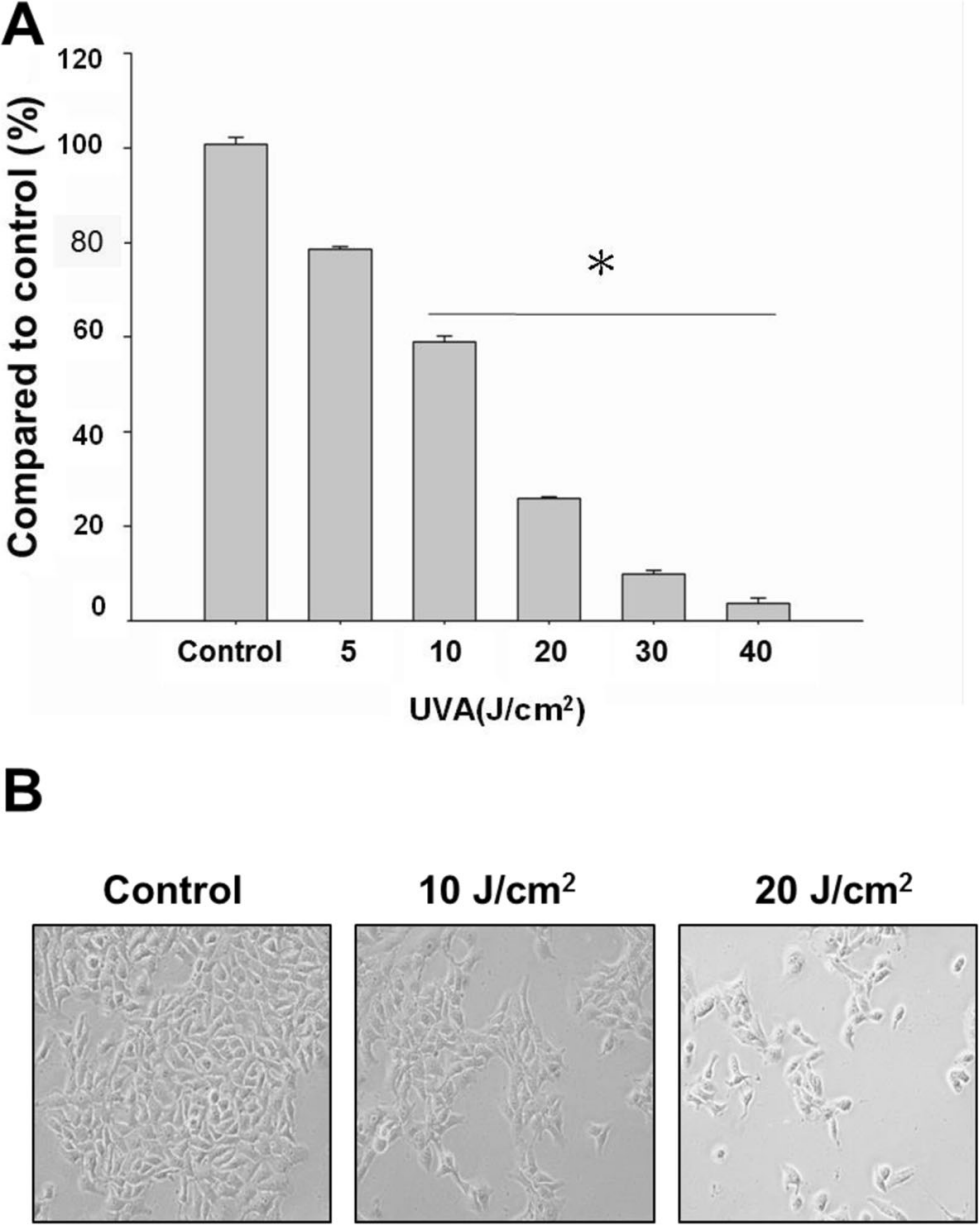
Evaluation of UVA-irradiation on the cellular viability of ARPE-19 cells. **a** MTT assay demonstrated that the viable cell number of ARPE-19 cells was suppressed by UVA radiation. **b** Morphological changes of ARPE-19 cells following UVA exposure. The results shown are three independent experiments (^#^ *p* < 0.05, *p < 0.001 compared with the control)

To examine the effect of UVA irradiation, ARPE-19 cells were exposed to various doses of UVA at 0, 5, 10, 20, 30 and 40 J/cm^2^. Cellular viability was then determined by MTT assay after a 24 h incubation. The results indicated that increasing doses of UVA radiation decreased ARPE-19 cell viability (Fig. 1a). The morphological changes of the cells exposed to UVA radiation were observed with inverted light microscopy. The growth of non-irradiated cells appeared compact and smooth; on the other hand, the cells treated with UVA irradiation above 10 J/cm^2^ had decreased cell populations compared with their counterparts (Fig. 1b). The cell populations treated with 30 and 40 J/cm^2^ UVA irradiation were reduced beyond the ability to appreciate them visibly, and thus are not presented.

### UVA induced apoptosis of ARPE-19 cells

**Fig. 2:**
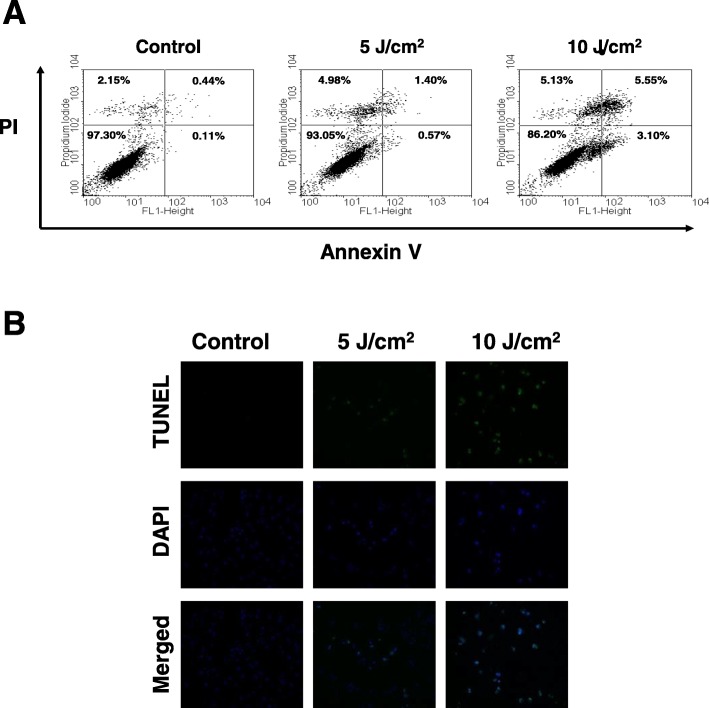
UVA-induced apoptosis of ARPE-19 cells. **a** Detection of apoptotic ARPE-19 cells by Annexin V / PI analysis following UVA irradiation (0, 5 and 10 J/cm^2^). **b** Detection of apoptotic cells by TUNEL and DAPI staining following UVA irradiation (0, 5 and 10 J/cm^2^)

To detect the cytotoxic effect of UVA irradiation on the induction of apoptosis, ARPE-19 cells were exposed to 5 J/cm^2^ and 10 J/cm^2^ UVA radiation, incubated for 6 h, and then stained with Annexin V-FITC/PI for flow cytometry analysis. An apoptotic rate of 3.1% was observed among the ARPE-19 cells following a 6 h 10 J/cm^2^ UVA exposure, compared to the 0.11% apoptotic rate observed among the control sample (lower right quadrant of Fig. 2a). A late apoptotic cell rate of 5.55% was observed among the ARPE-19 exposed to 10 J/cm^2^ of UVA, compared to the 0.44% apoptotic rate observed among the controls (Upper right quadrant of Fig. 2a).

These data demonstrate that UVA irradiation induced the apoptosis of ARPE-19 cells. To further validate the UVA-induced apoptosis of ARPE-19 cells, TUNEL/DAPI staining was performed to provide visual confirmation. UVA-induced massive apoptotic bodies were noted in ARPE-19 cells after exposure to 5 J/cm^2^ and 10 J/cm^2^ UVA radiation (Fig. 2b). These results clearly indicate that exposure to UVA radiation significantly induces the apoptosis of ARPE-19 cells, as determined by flow cytometric measurement and observation by fluorescence microscopy.

### Proteomic analysis to identify changes in protein profile after UVA radiation of ARPE-19 cells

**Fig. 3:**
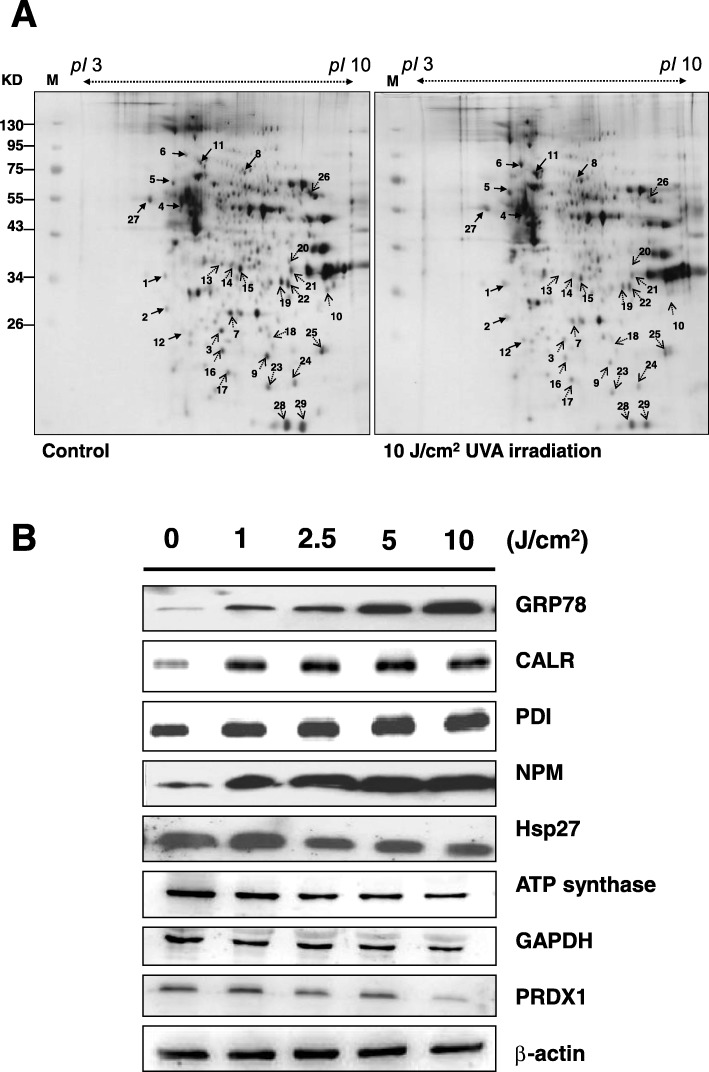
Identification of the differentially expressed proteins between the control and UVA irradiated ARPE-19 cells by 2-DE. **a** ARPE-19 cells irradiated with 10 J/cm^2^ UVA, followed by harvesting the cells and cell lysates. A total of 300 μg protein was then subjected to 2-DE and visualized by CBR staining. PDQuest image analysis software (Bio-Rad) was employed to explore differential expression through differential protein spotting. **b** Validation of identified selected proteins from 2-DE by western blot analysis. Some identified proteins include GRP78, CALR, PDI, NPM, Hsp27, ATP synthase, GAPDH, and PRDX1. β-actin was used as the loading control

Both ARPE-19 cells exposed to 10 J/cm^2^ UVA irradiation and unexposed cells were harvested. The proteins were extracted from cultured cells with cell extraction buffer. Following centrifugation, the supernatants were collected, and the proteins precipitated with 10% TCA/Acetone. The 2-DE maps of the non-irradiated ARPE-19 cells and 10 J/cm^2^ UVA irradiated ARPE-19 cells were compared to examine the effect of UVA irradiation. A total of 300 μg protein (*p*I 3–10) was loaded for the 2-DE experiments and visualized with CBR staining. PDQuest image analysis software (Bio-Rad) was employed to detect differential protein spotting (Fig. 3a).

Protein spots with more than a 1.5-fold difference were selected and further protein identification was performed using LC-MS/MS. Table 1 lists the identified proteins with their MASCOT scores, MS/MS matched sequences, apparent and theoretical Mw/pI, coverage, change in fold of expression level (up-regulation or down-regulation), cellular component, and protein function. Nine protein spots were up-regulated and 20 protein spots were down-regulated following UVA exposure.

**Table 1:** Proteins identification by LC/MS/MS

Pot no	Protein name	Accession.no	Calculate Mr/pI	Peptide matched	Sequence covered %	MASCOT score	Regulation (fold- change)	Cellular component	Protein function
1	Nucleophosmin	P06748	32.55/4.64	16	33	103	+ 2.64	Nucleus/ Cytoplasm	Cell proliferation,/apoptosis
2	Tropomyosin alpha-4 chain	P67936	28.5/4.67	4	18	61	+ 2.31	Cytoplasm	Cytoskeleton
3	Heat-shock protein 27	P04792	22.76/5.98	13	37	245	−1.92	Cytoplasm	Stress Response
4	Tubulin beta-4 chain	P04350	49.55/4.78	2	6	151	+ 2.72	Cytoplasm	Cytoskeleton
5	Protein disulfide-isomerase	P07237	57.08/4.76	7	15	89	+ 2.17	Endoplasmic Reticulum	Stress Response
6	78 kDa glucose-regulated protein	P11021	72.28/5.07	41	45	556	+ 4.19	Endoplasmic Reticulum	Stress Response
7	Triosephosphate isomerase	P60174	26.65/6.45	3	10	120	−2.08	Cytoplasm	Glycolysis
8	Bifunctional purine biosynthesis protein PURH	P31939	64.57/6.27	1	2	44	+ 2.64	Mitochondrion	Purine biosynthesis
9	Cell division protein kinase 10	Q15131	41.01/9.06	4	3	36	−3.08	Nucleus	Cell proliferation
10	L-lactate dehydrogenase A chain	P00338	36.66/8.44	12	23	145	−1.72	Cytoplasm	Glycolysis
11	Serum albumin precursor	P02768	69.32/5.92	31	11	194	+ 1.57	Cytoplasm	colloidal osmotic pressure of blood
12	Protein disulfide-isomerase A3	P30101	56.74/5.98	8	12	84	+ 2.72	Endoplasmic Reticulum	Stress Response
13	AH receptor-interacting protein (AIP)	O00170	37.64/6.09	2	6	49	−4.58	Cytoplasm	Protein folding
14	PDZ and LIM domain protein 1	O00151	36.04/6.56	4	13	59	−1.58	Cytoplasm	Cytoskeleton
15	PDZ and LIM domain protein 1	O00151	36.04/6.56	6	17	50	−1.56	Cytoplasm	Cytoskeleton
16	Heat-shock protein 27	P04792	22.76/5.98	83	59	874	−1.79	Cytoplasm	Stress Response
17	Heat-shock protein 27	P04792	22.76/5.98	25	31	121	−1.51	Cytoplasm	Stress Response
18	Proteasome subunit alpha type-2	P25787	25.99/6.92	16	38	111	−1.67	Nucleus/ Cytoplasm	Cell cycle,/apoptosis
19	Putative GTP-binding protein 9	Q9NTK5	44.71/7.64	11	8	38	−1.51	Nucleus/ Cytoplasm	ATP catabolic process
20	Fructose-bisphosphate aldolase A	P04075	39.39/8.3	22	43	211	−1.71	Cytoplasm	Glycolysis
21	Glyceraldehyde-3-phosphate dehydrogenase	P04406	36.03/8.57	17	34	67	−1.92	Cytoplasm	Glycolysis
22	Hydroxyacyl-coenzyme A dehydrogenase	Q16836	34.25/8.88	16	19	69	−1.52	Mitochondrion	Fatty acid metabolism
23	Alpha crystallin B chain	P02511	20.14/6.76	57	47	524	−1.85	Cytoplasm	Stress Response
24	Regulator of G-protein signaling 21	Q2M5E4	17.66/6.59	2	12	45	−1.72	Cytoplasm	Signal transduction inhibitor
25	Peroxiredoxin-1	Q06830	22.32/8.27	23	46	273	−1.56	Cytoplasm	Stress Response
26	ATP synthase	P25705	59.71/9.16	13	13	65	−1.58	Mitochondrion	Energy metabolism
27	Calreticulin	P27797	48.28/4.29	133	71	3246	+ 1.69	Endoplasmic Reticulum	Stress Response
28	Peptidyl-prolyl cis-trans isomerase A	P62937	18.23/7.68	8	23	129	−1.62	Cytoplasm	Protein folding
29	Peptidyl-prolyl cis-trans isomerase A	P62937	18.23/7.68	45	60	1160	−1.72	Cytoplasm	Protein folding

We classified these proteins into four groups according to their functions. The first group of proteins identified was involved in the stress response, cell proliferation, and apoptosis, including peroxiredoxin-1 (PRDX1), heat-shock protein 27 (Hsp27), protein disulfide-isomerase (PDI), calreticulin (CALR), 78 kDa glucose-regulated protein (GRP78), alpha crystalline B chain, cell division protein kinase 10, nucleophosmin (NPM), proteasome subunit alpha type-2, and regulator of G-protein signaling 21. The second group of proteins identified was related to glycolysis and energy metabolism, including triosephosphate isomerase, glyceraldehyde-3-phosphate dehydrogenase (GAPDH), L-lactate dehydrogenase A chain, fructose-bisphosphate aldolase A, and ATP synthase. The third group was related to cellular differentiation and the cytoskeleton, including tropomyosin alpha-4 chain, tubulin beta-4 chain, PDZ, and LIM domain protein 1. The fourth group was related to protein folding and other metabolism, including AH receptor-interacting protein (AIP), peptidyl-prolyl cis-trans isomerase A, hydroxyacyl-coenzyme A dehydrogenase, and albumin.

To validate the differential expression pattern obtained from the 2-DE maps, several identified proteins were investigated by western blotting using anti-CALR, GRP78, NPM, Hsp27, PDI, ATP synthase, PRDX1, and GAPDH specific antibodies. We observed four up-regulated proteins (CALR, GRP78, NPM, and PDI) and four down-regulated proteins (Hsp27, PRDX1, ATP synthase, and GAPDH) in the UVA-irradiated ARPE-19 cells, which is consistent with the exhibited expression pattern from the 2-DE maps (Fig. 3b).

### UVA irradiation induces the mitochondrial-related apoptotic pathway and caspase activation in ARPE-19 cells

UVA irradiation caused downregulation in the expression of ATP synthase, a mitochondrion-related protein involved in energy production. Mitochondria are essential to the intrinsic apoptotic pathway in which stressed cells engage in intracellular signaling that leads to the release of proteins from the mitochondrial intermembrane space. Mitochondrial dysfunction is therefore one characteristic of apoptosis and can be assessed through the change in mitochondrial transmembrane potential (ΔΨm). In the present study, we assessed the ΔΨm with JC-1 dye and achieved visualization via fluorescent microscopy.

**Fig. 4:**
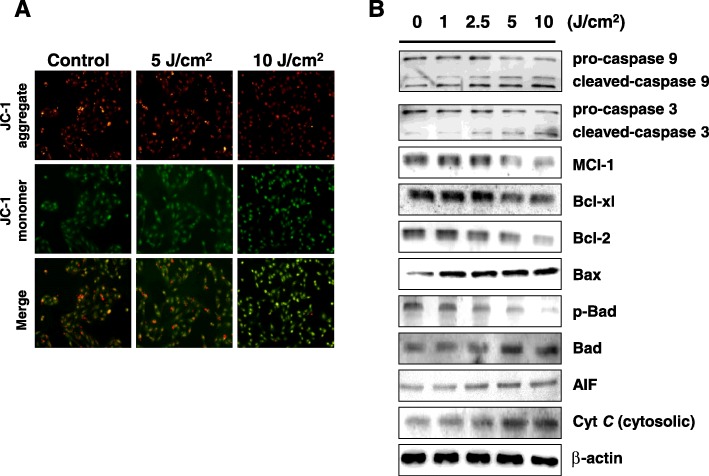
UVA-irradiation led to loss of mitochondrial membrane potential and induced mitochondria-related apoptotic pathways in the AREP-19 cells. **a** Cells were exposed to UVA (0, 5 and 10 J/cm^2^), stained with JC-1 dye, and imaged under fluorescent microscopy. **b** Western blotting data showed changes in pro-caspase-3, cleaved-caspase-3, pro-caspase-9, cleaved-caspase-9, Mcl-1, Bcl-xl, Bcl-2, Bax, Bad, p-Bad, AIF, and cytosolic cytochrome *C* expression in AREP-19 cells exposed to UVA. β-actin was used as an internal control

Staining with JC-1 permits for an assessment of mitochondrial health as it exhibits potential-dependent accumulation and indicates mitochondrial depolarization by a decrease in the red/green fluorescence intensity ratio. Red-fluorescent J-aggregates can be observed in healthy cells with high mitochondrial ΔΨm, while in apoptotic cells with low mitochondrial ΔΨm, the green-fluorescent monomeric form JC-1 is more apparent. The quantitative changes in transmembrane potential in this study were measured with a fluorescence plate reader to detect the emission of red and green fluorescence. In the present study, we assessed the color and pattern of JC-1 fluorescent staining to evaluate mitochondrial function following exposure of ARPE-19 cells to UVA radiation. We observed a markedly increased green fluorescence and reduction in red fluorescence following UVA irradiation (Fig. 4a). These results suggest that UVA irradiation led to a loss of ΔΨm and increased mitochondrial dysfunction.

To verify whether the mitochondrial-related apoptotic pathway is involved in UVA-induced apoptosis, we further detected several apoptotic-related mitochondrial proteins, including cytosolic cytochrome *C*, AIF, Mcl-1, Bcl-2, Bcl-xl, Bax, Bad, and p-Bad. The protein expression levels of cytosolic cytochrome *C*, AIF, Bax, and Bad were significantly increased in ARPE-19 cells following UVA irradiation. On the contrary, the protein expression levels of Mcl-1, Bcl-2, Bcl-xl, and p-Bad were significantly decreased following UVA exposure (Fig. 4b).

Caspase-3 and caspase-9 are known to be involved in cell apoptosis. We further investigated whether caspase activation was involved in UVA-induced apoptosis. In Fig. 4b, the western blotting results demonstrate that the expression levels of inactive pro-caspase-3 and pro-caspase-9 were decreased in the UVA-irradiated ARPE-19 cells while the expression levels of cleaved caspase-3, resembling active 17 kDa proteolytic fragments, and cleaved caspase-9, resembling active proteolytic 37 kDa fragments, were increased following UVA-irradiation.

### UVA radiation induces the ER stress-related pathway in APRE-19 cells

**Fig. 5:**
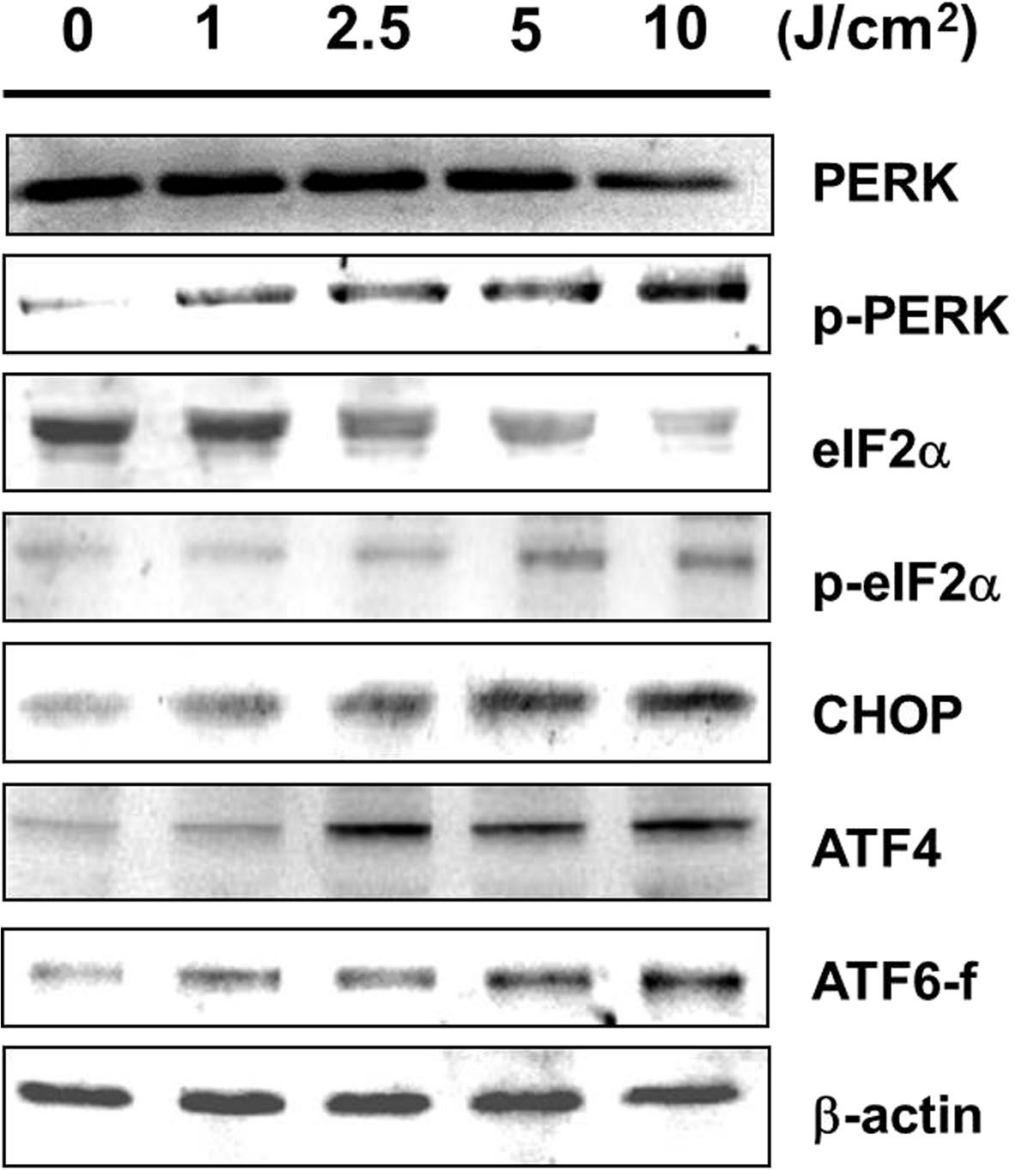
UVA-irradiation stimulated factors of the ER stress-mediated apoptotic pathway. The changes in ER stress sensors PERK, p-PERK, e-IF2, p-eIF2, ATF4, CHOP, and ATF6 fragment were investigated by western blot analysis. β-actin was used as the internal control

We used western blot to verify regulators of ER stress sensors, including PERK, ATF6, GRP78, PDI, and CALR. The expression of ER chaperones GRP78, PDI, and CALR following UVA-irradiation were up-regulated in a dose dependent fashion (Fig. 3b). Up-regulation was also noted for p-PERK and p-eIF2 (Fig. 5). A downstream signal of the PERK-eIF2 pathway, ATF4, was also upregulated following UVA-irradiation of APRE-19 cells, as was CHOP, an ER stress-induced nuclear protein (Fig. 5).

## Discussion

AMD is a leading cause of severe visual loss and blindness among the elderly population of the developed world [34]. The impairment of RPE cell function and degeneration of RPE cells are early and crucial events in the molecular pathology of AMD [2–5]. Oxidative damage is thought to play a critical role in the pathogenesis of AMD [8–11], and treatment with antioxidants has been demonstrated to reduce the progression of the pathology [13]. Overexposure to UV radiation, which is released by the sun and by other artificial sources such as tanning beds and sun lamps, causes the production of reactive oxygen species (ROS) that may cause oxidative damage to RPE cells [11] and contribute to the onset of AMD [28].

Our study investigated the cytotoxic effect of UVA radiation on ARPE-19 cell as examined by MTT assay, flow cytometry, fluorescent staining assays, proteomic analysis, and western blot analysis. As expected, ARPE-19 cells underwent cell apoptosis following UVA exposure. Flow cytometric analysis demonstrated that UVA radiation induces both early and late apoptosis. We also used comparative proteomic analyses to detect changes in the protein expression profiles of ARPE-19 cells exposed to UVA light. Some of these proteins such as CALR, GRP78, NPM, Hsp27, PDI, ATP synthase subunit alpha, PRDX1, and GAPDH are anti-proliferative, induce apoptosis, and provide protection against oxidative-stress.

We used several validation methods to prove that UVA irradiation induced apoptosis. We used TUNEL/DAPI staining to detect apoptotic signaling cascades as well as confirm UVA-induced apoptosis of ARPE-19 cells (Fig. 2).

Change in mitochondrial transmembrane potential (ΔΨm) is a critical coordination event of apoptosis. Mitochondrial ΔΨm change-induced pro-apoptotic events include the release of cytochrome *C*, which activates caspase-9 and in turn caspase-3, and the suppression of Bcl-2, Mcl-1, and Bcl-× 1 expression [35]. In our study, JC-1 staining revealed that UVA exposure led to a loss of mitochondrial ΔΨm. Following UVA exposure, western blot analysis revealed that the ARPE-19 cells had increased expression of pro-apoptotic proteins such as cytosolic cytochrome *C*, cleaved-caspase-3, cleaved-caspase-9, Bad and Bax, and decreased expression of anti-apoptotic factors Mcl-1, Bcl-2, Bcl-xl, and p-Bad. These results indicate the caspase-3, caspase-9, and mitochondria-mediated apoptotic pathways were involved in the UVA-induced apoptosis of ARPE-19 cells.

In the present study, 2-DE and western blot analyses indicated that the expression levels of ER chaperones GRP78, PDI, and CALR were increased after UVA-irradiation. Moreover, the expression levels of GRP78, CALR, and PDI increased in a dose-dependent manner with UVA exposure (Fig. 3b). The altered protein expression levels of GRP78, PDI, and CALR also suggest that the ER stress-induced apoptotic pathway was activated upon UVA-irradiation of ARPE-19 cells.

The accumulation of unfolded proteins in the ER causes the release of transmembrane ER proteins involved in inducing the unfolding protein response. In normal cells, GRP78 binds to transmembrane ER signaling proteins like PERK, IRE1α, and ATF6 [36–38] and activates them upon accumulation of unfolded proteins. PERK phosphorylates eukaryotic initiation factor 2 (eIF2α) to promote translation of activating transcription factor 4 (ATF4) and pro-apoptotic transcription factor CHOP [39]. However, up-regulation of GRP78 can help prevent cell death due to ER stress [40] through the inhibition of cytochrome *C*-mediated caspase activation [41].

We also analyzed the PERK-eIF2α-ATF4-CHOP signal pathway and another UPR sensor ATF6 to confirm the role of ER stress in UVA-induced apoptosis. The expression of p-RERK, p-eIF2α, ATF4, CHOP, and ATF6-f expanded significantly following UVA-irradiation (Fig. 5), suggesting the involvment of the PERK-eIF2α-ATF4-CHOP signal pathway and ATF6 due to the ER stress induced by UVA irradiation. In addition to confirming the role of ER stress in UVA-induced APRE-19 apoptosis, these results may also suggest that GRP78 serves as a therapeutic target in AMD.

We also used comparative proteomic analyses to detect changes in the protein expression profiles of ARPE-19 cells exposed to UVA light. LC-MS/MS analysis identified 29 differentially expressed proteins (9 up-regulated and 20 down-regulated) in the ARPE-19 cells exposed to 10 J/cm2 of UVA light. These proteins were classified into four groups: (1) stress response, cell proliferation, and apoptosis; (2) energy metabolism; (3) cell differentiation and cytoskeleton; (4) protein folding and other metabolism. The change in expression of these proteins was verified by western blot.

The first group of proteins identified was involved in the stress response, cell proliferation, and apoptosis, including peroxiredoxin-1 (PRDX1), heat-shock protein 27 (Hsp27), protein disulfide-isomerase (PDI), calreticulin (CALR), 78 kDa glucose-regulated protein (GRP78), alpha crystalline B chain, cell division protein kinase 10, nucleophosmin (NPM), proteasome subunit alpha type-2, and regulator of G-protein signaling 21. In a prior study investigating proteome changes in a rat model of UV-induced AMD, Kraljević Pavelić et al. also observed the involvement of crystallins [42]. They specifically detected the involvement of Gamma-crystallin D, which when irradiated leads to structurally specific modifications and precipitation via side chain damage, polypeptide crosslinking, or fragmentation by way of amorphous aggregates and amyloid fibers [43]. Crystallins also have aromatic residues which undergo thermal and photochemical reactions when exposed to UV light. These reactions have been suggested to impair the folding and stability of crystallin and contribute to cataractogenesis [44]. The alpha crystalline investigated in the present study is a particularly important structural protein of the lens [45, 46] and has been suggested to play a protective role against oxidative stress and retinal degeneration [47].

The second group of proteins identified was related to glycolysis and energy metabolism, and included triosephosphate isomerase, glyceraldehyde-3-phosphate dehydrogenase (GAPDH), L-lactate dehydrogenase A chain, fructose-bisphosphate aldolase A, and ATP synthase. Kraljević Pavelić et al. also observed that UV-radiation caused severe disruption of retinal metabolism, reducing metabolic activity and deregulating energy homeostasis, and detected a down-regulation of glycolytic enzymes, ATP production, and the citric acid cycle [42]. The retina has highest metabolic rate of all the tissues in the body [48] and over 50% of its ATP is derived through glycolysis [49]. Thus, our findings indicating UVA irradiation’s effect on the expression of glycolytic proteins may help support the notion that UV irradiation can proceed through hampering retinal cells’ ability to produce energy to cause the photoreceptor dysfunction and death characteristic of AMD.

The third group of differentially expressed proteins was related to cellular differentiation and the cytoskeleton, and included tropomyosin alpha-4 chain, tubulin beta-4 chain, PDZ, and LIM domain protein 1. The cytoskeleton plays an important role in vision. Some of its many and diverse functions include active transport, photoreceptor maintenance, and protein trafficking [50]. Narimatsu et al. demonstrated that light exposure works through oxidative stress to disrupt the actin cytoskeleton of RPE in mice [51]. Kraljević Pavelić et al. showed that in the rat retina UVB exposure proceeds through the activation of Src kinase to remodel and disintegrate the cytoskeleton [42], and Cachafeiro et al. suggested that photoreceptor cell death in AMD is caused by the light exposure-induced activation of Src kinase in the eye-cup and neural retina [52].

The fourth group of differentially expressed proteins observed in this study was related to protein folding and other metabolism, including AH receptor-interacting protein (AIP), peptidyl-prolyl cis-trans isomerase A, hydroxyacyl-coenzyme A dehydrogenase, and albumin. Kraljević Pavelić et al. also noted greater levels of serum albumin in rat retinas exposed to UV radiation [42]. Moreover, Nicolas et al. suggested that increased albumin may serve as a biomarker for UV-induced pathological change after observing greater levels of albumin in the retinas of monkeys with early onset macular degeneration than healthy monkeys [53].

The UVA dosages investigated in this study have physiological relevance in terms of both sun exposure and ophthalmological clinical treatment. After investigating the lethal dose of UVA radiation, we used doses of 5 and 10 J/cm^2^ for the experiments conducted in this study. At peak sunlight between noon and 1:15 p.m. at a latitude of 11 degrees north of the equator, the UVA radiation has been measured to be as high as 6.59 mJ/cm^2^ [54]. Thus, an exposure of only 12.6 min under these conditions would cumulatively equate to the lower (5 J/cm^2^) exposure used in this study. It is also possible that lesser exposures may accumulate and affect vision over time. While there is scant evidence for the dosimetric impact of radiation, several studies have found cumulative dose to be associated with visual acuity and blindness [55], as well as ocular structure and treatment toxicity [56].

Another condition in which humans may be exposed to UVA is through iatrogenic Mean*s. UVA* is now utilized clinically in the form of cross-linking (CXL). Combined UVA-riboflavin CXL has been increasingly used in the treatment of primary and secondary ectasia, such as keratoconus. One study used a total cumulative dose of 7.2 J/cm^2^ UVA irradiation per eye over an 8 min duration [57]. However, this procedure raises concerns regarding the potential hazards of UVA to the anterior segment, and especially to the retina which is more sensitive to UVA light than light of other wavelengths [58, 59]. A prospective three-year study showed that corneal collagen CXL induced no iatrogenic macular toxicity as per optical coherence tomography [60]. However, a more recent study disclosed that most of the patients who had been continuously exposed to UVA light during CXL therapy experienced slight changes in central macular thickness and multifocal electroretinography parameters [61]. Light toxicity may begin early following exposure but remain undetectable [62]. Thus, more frequent evaluations over shorter intervals following UVA-riboflavin CXL are recommended.

## Conclusion

Our results indicate that proteomic analysis is a feasible method to explore and discover potential markers in ophthalmic disease and advance our understanding of the potent effects of UVA irradiation on ARPE-19 cells at the molecular level. The present study showed that UVA irradiation can significantly inhibit the proliferation of ARPE-19 cells. The unfolded protein stress caused by up-regulation of GRP-78 or downstream cascade proteins and mitochondrial dysfunction, cytochrome C release, and caspase-9 and caspase-3 activation all encourage cellular apoptosis. The results in our current study have not been reported yet. These findings are promising and warrant further study for the development of new therapeutic strategies against UVA exposure.

## Data Availability

The data used in this study are provided in the figures and tables. The materials used in this study are cited in the text and are commercially available.
